# Avian injury quantification using the Shoreline Deposition Model and model sensitivities

**DOI:** 10.1007/s10661-019-7922-1

**Published:** 2020-03-17

**Authors:** Meredith Amend, Nadia Martin, F. James Dwyer, Michael Donlan, Michael Berger, Veronica Varela

**Affiliations:** 1 Industrial Economics Incorporated, 2067 Massachusetts Avenue, Cambridge, MA 02140 USA; 2 Natural Resource Damage Assessment and Restoration Program, United States Fish and Wildlife Service, Alaska Regional Office, 1011 East Tudor Road, Mail Stop #361, Anchorage, AK 99503 USA

**Keywords:** Shoreline Deposition Model, Beached Bird Model, Deepwater Horizon, Oil spill, Damage assessment, Avian injury

## Abstract

Deposition models, such as the Shoreline Deposition Model (SDM) used to quantify nearshore avian injuries resulting from the 2010 *Deepwater Horizon* (DWH) oil spill, were developed to improve the estimates of nearshore avian mortality resulting from the release of oil into coastal and marine environments. Unlike earlier approaches to injury quantification, such as simple counts of carcasses on the shoreline, a modeling approach allows trustees to evaluate the precision of their estimate (i.e., to develop a confidence interval) and can inform decision-making and the likely utility of additional primary data collection activities through sensitivity analyses. In this paper, we rely on published literature, actual DWH data, and a deposition model simulation to evaluate how different model inputs and assumptions can affect the accuracy and precision of model results. We find that the precision of deposition models is strongly affected by the length of time between subsequent shoreline searches, the underlying magnitude of carcass deposition, carcass persistence probabilities, and carcass detection probabilities. In addition, the accuracy of deposition model results may be affected by natural fluctuations in deposition rates. Given these findings, we recommend that natural resource trustees include an evaluation of future model uncertainty as part of their initial data collection efforts. This will allow them to deploy resources in a way that maximizes the utility of future deposition model results. We also identify several factors that *do not* need to be assessed immediately following a spill event, thereby potentially freeing resources for more time critical data collection efforts.

## Introduction

Following oil spills or other releases of hazardous substances, federal and state agencies may act as trustees on behalf of the public to assess natural resource injuries and recover damages for such injuries under the Oil Pollution Act (OPA; 33 U.S.C. §§ 2701–2761) or the Comprehensive Environmental Response and Liabilities Act (CERCLA; 42 U.S.C. §§ 9601–9675). On April 20, 2010, the *Deepwater Horizon* (DWH) oil spill began after a mobile drilling unit exploded, caught fire, and eventually sank in the Gulf of Mexico. For a period of almost 90 days, oil continued to spill into the Gulf of Mexico until the well was capped on July 15, 2010. During that time, and for several months after the well was capped, natural resources were adversely affected by the oil spill. One of the impacted resources federal and state trustees assessed was birds (IEc 2015a).

Over the past several decades, a variety of approaches have been used to estimate the total number of birds impacted or killed by an oil spill. One of the earliest approaches relied on direct observations of the number of dead birds collected or the number of oiled birds observed along the shoreline (Greenwood and Keddie 1968). Other early methods modified these raw numbers with simple multipliers. For example, after the 1970 Hamilton Trader oil spill, total avian deposition was estimated by multiplying the average number of carcasses and oiled birds observed during shoreline surveys by the length of unsearched shoreline with similar characteristics within the spill-impacted area (Hope Jones et al. 1970). Page and Carter (1986) took into account the potential for birds to be lost at sea when estimating the number of birds that were debilitated or killed as a result of the San Joaquin Valley crude oil spill in California.

However, these early methods did not account for several other factors that influence estimates of the total number of spill-impacted birds following a discharge event. Modeling approaches developed in the late 1980s and 1990s accounted for gaps in search area and losses at sea, but also for potential losses along the shoreline due to scavenging or decomposition (referred to as “carcass persistence”) and the number of birds potentially missed by wildlife searchers (referred to as “searcher efficiency” or “detection probability”) (Byrd et al. 2009; Ford and Zafonte 2009; Ford 2006; Fowler and Flint 1997; Van Pelt and Piatt 1995; and Piatt and Ford 1996). This carcass deposition modeling approach is the current state of the art and has been applied to several spills, including the *Apex Houston*, the *Jacob Luckenbach*, *Citrus,* and the *Cosco Busan* (Page et al. 1990; Ford et al. 1996; Flint et al. 1999; Ford et al. 2006, 2009).

The most recent application of these methods was the use of the Shoreline Deposition Model (SDM) to quantify nearshore avian injuries resulting from the 2010 DWH oil spill (IEc 2015a). The nearshore environment includes the portion of the northern Gulf of Mexico that is within 40 km of the shoreline. Avian mortality also occurred in offshore waters greater than 40 km from the shoreline, but that was addressed using a separate methodology (IEc 2015b). Over 7500 dead and debilitated birds were collected from shorelines after the DWH spill. The SDM utilized this information, along with area-specific factors to account for searcher efficiency, carcass persistence, and gaps in search effort, to derive daily avian deposition estimates for the northern Gulf of Mexico coastline.

The primary goal of avian injury quantification using deposition models is to produce an accurate estimate of nearshore avian mortality. Current deposition models fit this need. However, because deposition models rely on sampling design and extrapolation, interpretation of the results allows for assessment of sampling error. An analysis of different sources of model uncertainty can provide natural resources trustees with a foundation for deciding how to allocate finite data collection resources. Understanding the precision of model results can also help to inform the likely utility of additional primary data collection activities. In this paper, we investigate some of the major sources of uncertainty in the SDM and other deposition models. Through this analysis, we highlight components of uncertainty that are impacted by primary data collection choices made by trustees during the spill. This analysis relies on a review of published literature, a deposition model simulation and an analysis of actual data collected for DWH nearshore avian injury quantification.

## SDM and deposition model approach

The SDM and other deposition models use the number of collected birds, along with area-specific information on *searcher efficiency* and *carcass persistence*, to derive an estimate of actual avian deposition. In some cases, it may be possible to obtain estimates of these parameters from published literature. However, several studies have found that these parameters can vary due to a range of site-specific factors, including beach type, weather, tidal activity, scavenger activity, and carcass size, etc. (Byrd et al. 2009; Varela and Zimmerman 2016; Zimmerman and Varela 2016). In the case of DWH, site-specific field studies were conducted to estimate searcher efficiency and carcass persistence values that were specific to the northern Gulf of Mexico.

Using these inputs, avian deposition is estimated for a discrete *length* of shoreline (i.e., a shoreline “segment”). Typically, shoreline segments are not searched daily, and because the number of collected birds from a given search represents deposition from every day since the previous search of a shoreline segment, the model calculations are completed for individual *search intervals*—defined as the time period between two subsequent searches of the same shoreline segment. The calculated daily deposition rate (birds per kilometer) for a given *search interval* is then applied to each day in the interval.

For each day of the modeled time period, deposition rate estimates from individual shoreline segments with similar characteristics (e.g., geographic location and habitat type) are averaged and applied to the total length of shoreline in a defined *extrapolation area* (IEc 2015a). Extrapolation areas are used to group shoreline segments that should experience similar rates of avian deposition (i.e., have similar habitat types and are subject to similar wind and wave action); because they are a modeling construct, their boundaries can be altered throughout the modeling process (IEc 2015a). Deposition models make several assumptions in order to derive an estimate of actual avian deposition from the *number of collected birds*. Common assumptions include (1) wildlife searchers have a probability of finding a bird on the shoreline that is equal to the *searcher efficiency* factor, (2) the proportion of carcasses that persist until the next search will be equal to the *carcass persistence* probabilities, and (3) the daily deposition rate of carcasses and injured birds is constant between searches (IEc 2015a; Ford et al. 2009; Page et al. 1990).

To illustrate the equations underlying the SDM and deposition models, imagine a shoreline segment that is searched first on Monday and again on Friday (i.e., a 4-day search interval). The variable “*n*” will represent the number of days until the next search (i.e., Tuesday will be represented by “*n*_3_” because there are 3 days until the next search—Wednesday, Thursday, and Friday; and Friday will be represented by “*n*_0_”). The number of birds we expect to collect on Friday can be represented by equation 1:

1 $$ {N}_C=E\ast \sum \limits_{n=3}^0\left({D}_n\ast {P}_n\right) $$

where,

*N*_*C*_: the number of carcasses collected from the specified shoreline segment on Friday (*n*_0_).
*E*: the applicable searcher efficiency value
*D*_*n*_: the number of birds deposited on day n
*P*_*n*_: the probability that a bird deposited on day n will persist until Friday (*n*_0_)

By making the assumption of constant daily deposition, *D*_*n*_ can simply be represented by *D* and equation 1 can be simplified to equation 2:

2 $$ {N}_C=E\ast D\ast \sum \limits_{n=3}^0\left({P}_n\right) $$

When estimating nearshore avian mortality for a spill, the daily deposition rate is unknown, while values can be generated for *N*_*C*_, *E*, and *P*_*n*_. Therefore, by solving for *D* and adding a few additional parameters (described below), we get equation 3, which is used in the SDM and was published in Ford et al. 1987 and Page et al. 1990.

3 $$ D=\frac{N_C-{N}_O}{L\cdot E\cdot \sum \limits_{n= SI}^0{P}_n} $$

where,

*D*: the daily deposition rate (birds per kilometer) for a shoreline segment within a search interval (SI); in our example, between Monday and Friday.
*N*_*C*_: the number of carcasses collected during a search of the specified shoreline segment
*N*_*O*_: the number of “old” carcasses collected during a search that were actually deposited, but not collected, during the previous SI
*L*: the length, in kilometers, of the applicable shoreline segment (added to normalize *D* to birds per kilometer (rather than birds per segment)
*E*: the applicable searcher efficiency factor
$$ \sum \limits_{n= SI}^0{P}_n $$
the sum of applicable persistence probabilities, where *n* is decremented from SI to 0

**Fig. 1:**
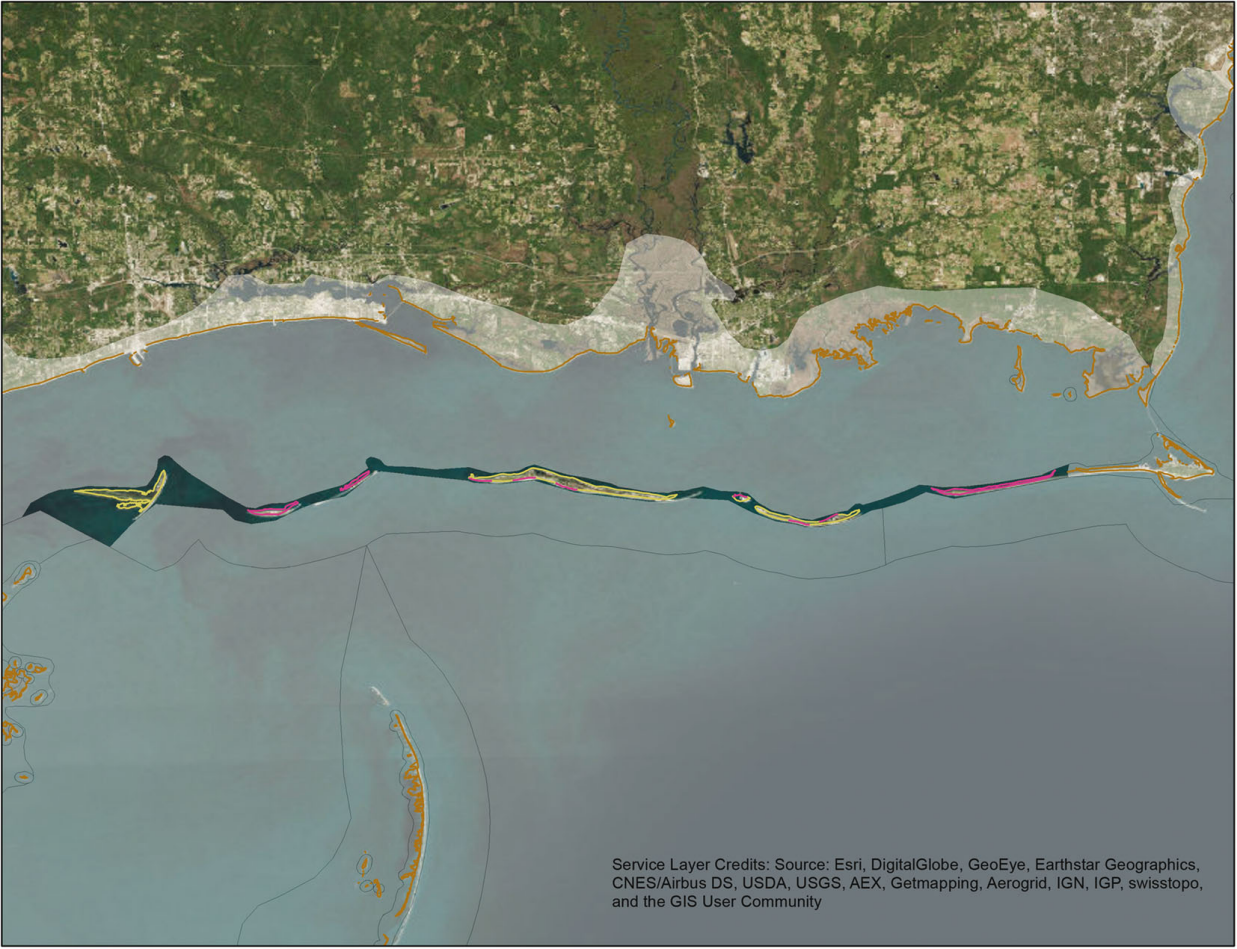
Example of a DWH SDM extrapolation calculation done for an extrapolation area off of the Mississippi–Alabama coast for August 7, 2010. An extrapolation area off of the Mississippi–Alabama coast is illustrated above and contains a total of 114 shoreline segments. One hundred and one of the segments are dominated by beach habitat and 13 are dominated by marsh habitat. For this day, sufficient data were available for the SDM to calculate deposition rates (birds/km) for 27 of the 114 shoreline segments (the segments highlighted in pink above). Of these 27 segments, 25 were classified as beach habitat. To calculate the total number of birds that deposited on beach habitat in this extrapolation area on August 7, the average of these 25 deposition rates was calculated (0.13 birds/km) and then multiplied by the total length of beach habitat shoreline in that extrapolation area (116.5 km). As such, the SDM estimates that a total of 15.15 birds were deposited on beach shoreline in this extrapolation area on August 7. The remaining two segments were classified as marsh habitat. The average of their deposition rates was 0 (because no birds were collected from those segments during the associated shoreline searches). As such, the SDM estimates that no birds were deposited on marsh shoreline in this extrapolation area on August 7

Using the process above, deposition models generate daily deposition estimates for each segment that meets user-defined data requirements within the model time period (e.g., if the user decides to use a maximum search interval of 5 days, only segments searched at least this frequently will be included in the model). For each day of the modeled time period, daily deposition rates for individual shoreline segments of the same habitat are then averaged by the model. This average daily deposition rate is then applied to the length of shoreline within a user-defined extrapolation area to generate an estimate of total avian deposition in that extrapolation area for that day. An example of this process is described in Fig. 1. Finally, the deposition model sums daily estimates from all extrapolation areas to generate the final estimate of nearshore avian deposition for the modeled time period.

## Methods

To investigate the sensitivity of deposition model results to various inputs and assumptions, we rely on published literature, actual DWH data, and a deposition model simulation. Using the model simulation, we focus specifically on factors and assumptions that can impact *N*_*C*_, and, by extension, *D* and overall model results. For example, the model equations imply a deterministic relationship between the number of deposited birds and the number of birds that will be collected during shoreline searches. In reality, this relationship is probabilistic, and each bird has an independent probability of persisting until the next search equal to the carcass persistence probabilities as well as an independent chance of being detected during the next shoreline search equal to the searcher efficiency value. This can generate differing values of *Nc* even when the same underlying number of birds are deposited. The deposition model simulation explores how this fact, as well as natural deviations from constant deposition between searches, may affect model precision and accuracy. While additional variance can be introduced due to uncertainty associated with the estimates of the carcass persistence and searcher efficiency probabilities, the effect of those uncertainties on model accuracy and precision is not explored in this analysis. However, we provide context for this component in the “Discussion” section.

The deposition model simulation produces carcass deposition and model calculations for a theoretical 1-km stretch of shoreline that for simulation purposes is assigned a pre-determined level of carcass deposition (either 1 or 10 birds per day) over a 12-day time period. Deposition rates were selected to encompass the majority of deposition values that were observed following the DWH spill, and a 12-day time period was selected because it could be evenly divided into several different search frequencies that are typically used during data collection.

Within the simulation, each deposited bird has an independent probability of persisting until the next search date equal to the persistence probabilities in Table 1 (i.e., a bird deposited on a beach segment on day *n6* has a 43% chance of persisting until the day of the next search). Each bird also has an independent probability of being collected on the next search date equal to the searcher efficiency values in Table 1 (i.e., an 86% chance of being detected on a beach segment and a 43% of being detected on a marsh segment). These searcher efficiency and carcass persistence values are the beach habitat- and marsh habitat–specific values that were determined for the northern Gulf of Mexico and used in the DWH SDM analysis. These values fall within the range of searcher efficiency and carcass persistence values calculated for other oil spills, and therefore provide a representative case study (Ford et al. 1991a, b; Burger and Fry 1993; Van Pelt and Piatt 1995; Fowler and Flint 1997; Morrison 2002; Byrd et al. 2009; and Ford et al. 2009).

**Table 1:** DWH oil spill searcher efficiency and carcass persistence values used for the SDM (derived from Varela et al. 2015a, b)

	*n*, days until next search	Beach habitat	Marsh habitat
Searcher efficiency	–	0.86	0.43
Carcass persistence	0	1.00	1.00
	1	0.72	0.46
	2	0.61	0.31
	3	0.54	0.24
	4	0.49	0.20
	5	0.45	0.18
	6	0.43	0.16
	7	0.41	0.15
	8	0.39	0.14
	9	0.37	0.13
	10	0.36	0.13
	11	0.35	0.12

To examine a range of SIs, deposition magnitudes, searcher efficiency values, and carcass persistence values, the simulation was run using six different SIs, three pairs of searcher efficiency and carcass persistence values and two deposition rates. The six SIs were 1 day (i.e., 12 searches conducted within the simulated 12-day time period), 2 days (i.e., six searches conducted within the simulated 12-day time period), 3 days, 4 days, 6 days, and 12 days. The three sets of searcher efficiency and carcass persistence values were (1) high searcher efficiency and carcass persistence values (represented by the “Beach Habitat” values in Table 1), (2) low searcher efficiency and carcass persistence values (represented by the “Marsh Habitat” values in Table 1), and (3) low carcass persistence values with an increased searcher efficiency rate of 0.7. This rate was selected because it represents an improvement in searcher efficiency rates that can be achieved if two passes of a shoreline segment are made (Byrd et al. 2009). The two deposition rates analyzed were 1 bird per kilometer and 10 birds per kilometer.

The simulation was run 1000 times (i.e., 1000 “trials”) for each of the SIs within each of the four treatments to reveal the range of model results that could be generated from the same initial conditions. Within each trial, the number of carcasses that persist until, and are found on, the next search date represents the trial number of collected carcasses (*N*_*C*_). The trial *N*_*C*_ value is then inserted into equation 3 above to generate a trial deposition model result. For each trial with multiple searches during the 12-day modeled time period (i.e., all conditions except an SI of 12 days), results for each search were averaged and then applied across the total 12-day model time period to generate a single result per trial. The mean, standard deviation, and coefficient of variation (the standard deviation divided by the mean) of all model runs were calculated for each SI within each treatment.

The assumption of constant deposition between subsequent shoreline searches is inherent to equation 3 and is commonly assumed in shoreline deposition models. However, actual carcass deposition likely varies from day to day due to a variety of site-specific factors such as weather, the presence of live birds at risk of oiling, and the presence of oil. To test the importance of the constant deposition assumption, the model simulation was adjusted to deposit all of the birds on either the first day of the SI (i.e., the day right after the previous search), or the last day of the SI (i.e., the day of the current search). These deposition patterns were selected because they represent the two possible extremes, and so provide a measure of the maximum potential uncertainty introduced by the constant deposition assumption. The simulation was again run 1000 times for each combination of SI, searcher efficiency, carcass persistence, and deposition rate. Simulated model results were then compared to the known number of deposited birds (i.e., 12 or 120).

## Results

The model simulation informs the sensitivity of deposition model results to different search intervals, under scenarios that capture high and low deposition rates, searcher efficiency values, and carcass persistence values. Summary statistics of the simulation results for the six different SIs and three sets of searcher efficiency and carcass persistence values are shown in Table 2 for a deposition rate of 10 birds per kilometer. For each SI in all three treatments, the average model result is not significantly different than the total known number of deposited carcasses (i.e., 120 birds). As previously published, this is expected so long as the assumption of constant deposition holds true (Ford et al. 1991a; Entrix 2008).

**Table 2:** Summary statistics for deposition model simulations (mean SDM result (*x*), standard deviation (*σ*) and coefficient of variation (CV)) with different SIs under the three treatments using a deposition rate of 10 birds per kilometer

	High searcher efficiency and carcass persistence			Low searcher efficiency and carcass persistence			Low carcass persistence and a searcher efficiency of 70%		
Search interval	*x*	*σ*	CV	*x*	*σ*	CV	*x*	*σ*	CV
1-day	119.86	4.46	3.7%	119.76	12.35	10.3%	120.05	7.14	5.9%
2-day	120.05	6.13	5.1%	120.45	15.49	12.9%	120.15	9.64	8.0%
3-day	119.89	7.38	6.2%	120.13	18.34	15.3%	120.52	11.91	9.9%
4-day	120.42	8.03	6.7%	119.76	20.21	16.9%	120.07	13.25	11.0%
6-day	120.35	9.82	8.2%	118.68	23.21	19.6%	119.99	15.67	13.1%
12-day	120.34	11.39	9.5%	118.86	29.16	24.5%	120.07	20.92	17.4%

**Fig. 2:**
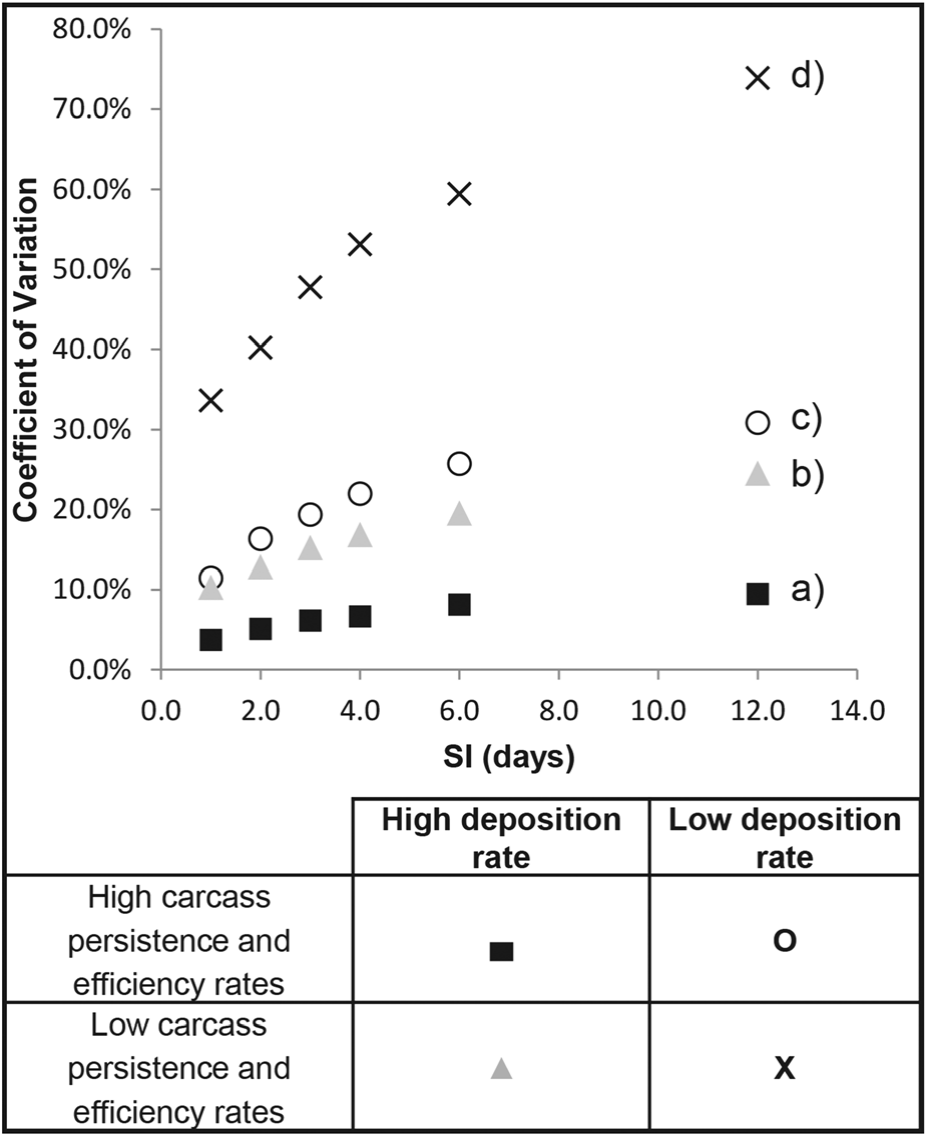
A plot of the coefficient of variance (*y*-axis) against the length of the SI (*x*-axis) under four different scenarios: (a) high persistence and efficiency values and a high deposition rate, (b) low persistence and efficiency values and a high deposition rate, (c) high persistence and efficiency values and a low deposition rate, and (d) low persistency and efficiency values and a low deposition rate

Our results also show that the standard deviation and the coefficient of variation (*σ*/*x*) associated with a given result increase directly with the length of the SI and indirectly with searcher efficiency and carcass persistence probabilities (Fig. 2). While the deposition model continues to produce an accurate estimate of avian deposition on average (i.e., the average model result generated is consistent with the known level of carcass deposition), the precision of that estimate declines (i.e., the variance between individual model runs increases) as the length of the SI increases and as searcher efficiency and carcass persistence decrease. This relationship is strongly influenced by the magnitude of deposition, as demonstrated by the differences between scenarios “a” and “c” and “b” and “d” in Fig. 2. This decline in precision is expected given the smaller sample size at lower deposition rates.

In order to provide context for natural resource injury estimates, confidence intervals are frequently calculated to account for uncertainty in results. The variance described in Table 2 is one source of uncertainty that would need to be accounted for in a confidence interval associated with a deposition model analysis. The larger variance associated with longer SIs and lower deposition rates could lead to a preference for shorter search intervals in order to limit the magnitude of the deposition model result’s confidence interval.

In addition, comparing the treatments that utilize low carcass persistence values with either a low searcher efficiency rate of 43% or a higher searcher efficiency rate of 70% reveals some of the effects that different sampling regimes can have on model uncertainty. By switching from one pass of a shoreline segment to two the uncertainty associated with each search interval is decreased. However, given that total resources are likely to be limited, it is more realistic to compare the 70% uncertainty values to search intervals that are half as long. This assumes that a doubling of search intensity would lead to a halving of search frequency. However, even this comparison reveals that the gain in precision from increased search intensity is worth the loss in search frequency.

**Fig. 3:**
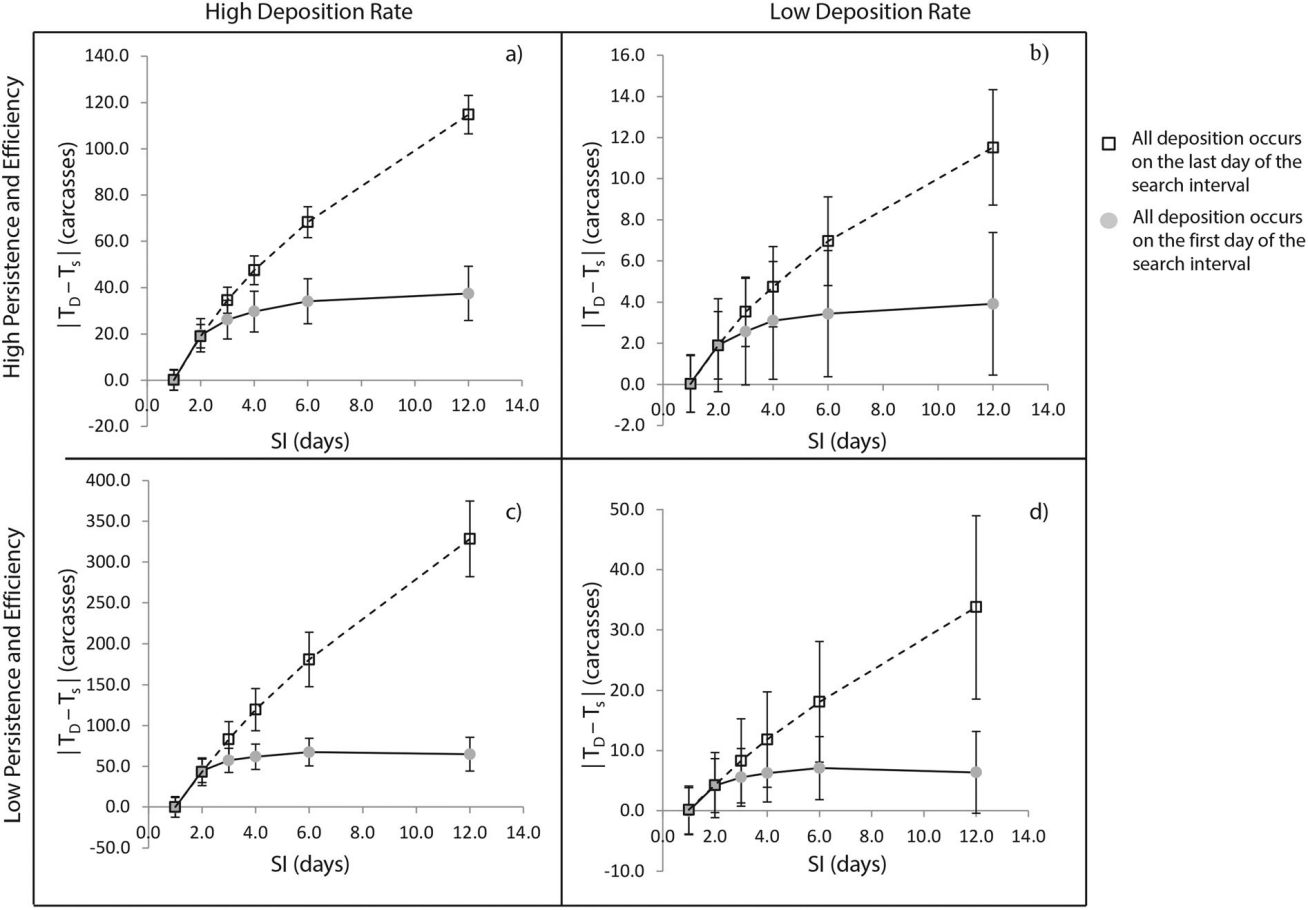
A plot of the maximum potential absolute difference between known avian deposition (*T*_*D*_) and the average calculated amount of avian deposition (*T*_*s*_) (*y*-axis) against SI (*x*-axis) under four different scenarios: (**a**) high persistence and efficiency values and a high deposition rate, (**b**) high persistence and efficiency values and a low deposition rate, (**c**) low persistence and efficiency values and a high deposition rate, and (**d**) low persistency and efficiency values and a low deposition rate. Circles display the maximum potential magnitude of underestimation caused by “early” deposition, and squares display the maximum potential magnitude of overestimation due to “delayed” deposition. Error bars representing one standard deviation are also shown

Additional model simulations were used to analyze the impact of depositing all of the birds on either the first day of the SI or the last day (which corresponds to the day of the current search). This allowed us to evaluate the maximum potential uncertainty introduced by the model’s constant deposition assumption. If all of the birds are deposited on the first day of the SI, the model simulation underestimates total deposition, because the deposited carcasses are subject to more loss than is accounted for by the persistence value used in the model calculation. Because the model assumes constant deposition, it assumes that the number of collected birds (*N*_*C*_) comprises some portion of birds that were deposited on the day after the previous SI, some the day after that, and so on until the day of the current search. As the number of days until the next search decreases, the likelihood that a carcass will persist until the next search increases (Table 1). However, if all of the birds are actually deposited on the day immediately after the previous search, they all have an equally low probability of persisting until the next search. As a result, the simulated *N*_*C*_ is low, leading to an underestimation of daily deposition rates by the model. Comparatively, if all of the birds are actually deposited on the day of the current search, the model simulation overestimates daily deposition because the deposited carcasses are subject to less loss than is accounted for by the persistence value used in the SDM calculation. These birds all share an equally high probability of persisting until the next search, which leads to a higher *N*_*C*_ and overestimation of daily deposition rates by the model calculation. The result of this effect for the six different SIs and the four different treatments is shown in Fig. 3.

The results of this analysis indicate that the deposition model calculations can overestimate or underestimate actual avian deposition when natural deviations from constant avian deposition occur on shoreline segments. The maximum potential magnitude of overestimation or underestimation that may occur within model calculations is influenced by the magnitude of deposition (i.e., the number of deposited carcasses) and the length of search interval. This relationship seems to be independent of searcher efficiency and carcass persistence. Across treatments with the same searcher efficiency and carcass persistence rates (treatments “a” and “b” and “c” and “d” in Fig. 3), the percentage difference between the maximum potential magnitude of overestimation and underestimation for any one search interval is fairly constant.

Under the simulated conditions, the maximum potential magnitude of overestimation begins to exceed the maximum potential magnitude of underestimation when the search interval exceeds 2 days, which impacts the accuracy of deposition model calculations. In these extreme scenarios (i.e., all birds either deposited on the first or last day of the search interval), on average, the model calculations will tend to overestimate underlying avian deposition. Because the magnitude of overestimation can be predicted and is consistent across shoreline segments with the same searcher efficiency and carcass persistence values, this source of potential bias could likely be corrected for in deposition model calculations.

In practice, it is likely that daily deposition will fluctuate, reflecting a circumstance between the model assumption of constant deposition and the “bounding” scenarios of all deposition occurring on the first or last day of search intervals. For example, information from a sample of 13 segments that were searched for four consecutive days during the DWH spill (the longest time period that any segments were searched consecutively) suggests that, for this spill, deposition rates varied even over short time intervals. Table 3 below presents the number of carcasses that were collected from these segments on each consecutive day that they were searched. While carcass collections from consecutive daily searches are not the same as the underlying deposition rate (because they can still be impacted by searcher efficiency), they are directly related to deposition, and are the best available representation of the underlying deposition rate. While none of the segments shown in Table 3 appear to exhibit a constant deposition rate over the full 4-day time interval, neither did they exhibit the deposition patterns as extreme as those explored in the model simulation bounding scenarios described above.

**Table 3:** Number of carcasses collected each day from shoreline segments searched consecutively for 4 days

Segment	Day 1	Day 2	Day 3	Day 4
A	0	1	1	0
B	0	1	1	0
C	0	1	1	0
D	0	1	1	0
E	0	1	2	0
F	0	1	1	0
G	0	0	2	1
H	0	0	1	2
I	1	1	0	0
J	0	2	1	0
K	4	4	0	0
L	0	0	13	13
M	0	1	5	0

## Discussion

There are many considerations and decisions involved when using a SDM-type deposition model to estimate total nearshore avian mortality. These include quantifying data inputs such as the number of birds collected after a spill, estimating searcher efficiency and carcass persistence, identifying segment-specific search schedules, and defining extrapolation areas. A thorough evaluation of all the inputs, assumptions, and decisions required for deposition model calculations is outside the scope of this paper. This analysis focuses on a few key components and seeks to provide a framework from which natural resource trustees can evaluate how different site conditions and sampling approaches may impact model uncertainty, enabling them to more efficiently and effectively allocate resources during oil spill events to assess impacts to birds.

When natural resource damage assessment (NRDA) personnel identify the initial level of search effort, important factors to consider include the various habitat types impacted by the spill, the total length of potentially impacted shoreline, accessibility, and any funding or personnel limitations. As part of this initial evaluation NRDA personnel should use available literature to try and estimate what the relative searcher efficiency and carcass persistence rates might be for different impacted areas. Because areas with different characteristics will result in different levels of uncertainty, this assessment will allow trustees to deploy resources in a manner that minimizes uncertainty for a given level of effort/expenditure. This may result in an uneven distribution of resources between areas as well as differing search patterns. For example, if an area is likely to have a high searcher efficiency probability, resources should be focused on searching segments as frequently as possible. However, if searcher efficiency is expected to be low it may be more effective to increase search intensity.

If these factors cannot be estimated based on a site assessment or the literature, it may be best to set a regular search schedule with the shortest search interval allowable based on available personnel and funding. The shoreline segments searched as part of these initial efforts should focus on the areas that are most likely to receive early avian deposition. Data collected during these early searches can be used to refine data collection as the spill-impacted time period progresses. For example, carcass persistence studies could be conducted concurrent with early shoreline searches. Rather than collecting all encountered carcasses, some of the not visibly oiled, relatively fresh carcasses could be tagged by NRDA survey personnel, left in place, and then tracked for their persistence on subsequent surveys. Close coordination with wildlife response teams and oil cleanup crews would be required to prevent these teams from collecting the marked carcasses. Once the carcass persistence probabilities for the spill-impacted area have been assessed, the search schedule can be adjusted accordingly.

While not specifically examined in this paper, the precision of searcher efficiency and carcass persistence probabilities is another source of uncertainty that can be managed by NRDA personnel. Recall that these probabilities are affected by several site-specific factors, including beach type, weather, tidal activity, scavenger activity, and carcass size, etc. (Byrd et al. 2009; Varela and Zimmerman 2016; Zimmerman and Varela 2016). Primary data collection efforts that target these different factors can lead to more precise estimates that minimize this source of uncertainty. These studies can also be, and have been, conducted in the years following a spill event as long as careful consideration is given to match the original spill conditions as much as possible. This allows trustees to decide at a later date if this level of effort is warranted.

One of the largest sources of both uncertainty and accuracy is the overall magnitude and pattern of deposition for a spill event. This is also a component that cannot be influenced by factors under the trustees’ control. To reduce the risk of over- or underestimation, search intervals no longer than 2 days should be used. However, this is likely to be overly conservative and cost-prohibited. A less effort-intensive approach would be to search some segments daily and the others much less frequently. Then, the observed patter of deposition from the segments searched daily can be used to correct for any potential bias in the deposition model.

While the definition of extrapolation areas was not evaluated within this paper, it is another example of a model input that can be addressed after data collection has ended. Extrapolation areas are simply used to group together shoreline segments (and any unsearched shoreline between them) expected to experience similar levels of avian deposition. While the actual process of defining extrapolation areas is complex, these groups can be defined or redefined throughout the injury quantification process. As such, this decision does not need to be made during early spill efforts and a variety of options can be explored in deposition model results.

## Conclusions

The goal of avian injury quantification in the context of CERCLA and OPA is to produce an accurate estimate of avian mortality that is also reasonably precise (i.e., has an acceptably narrow confidence interval). Modeling approaches, such as the SDM, are currently the best approach to meet this goal while addressing multiple factors that impact the number of avian carcasses and debilitated birds collected or observed after a spill event. However, the ability for the SDM and deposition models to deliver accurate and precise estimates of avian injury is strongly linked to data collection procedures and associated modeling decisions.

The theoretically best approach to data collection would be to search the entire spill-impacted shoreline on every day of the spill-impacted time period. However, this approach is rarely an option during an oil spill event given effort and expenditure constraints, particularly for large spills like the 2010 DWH spill. For most spills, a subset of the total shoreline is searched at a frequency less often than once per day. In these cases, careful planning of search effort can improve the efficacy of an SDM-type analysis.

The analyses in this paper show how model inputs and assumptions can impact the accuracy and precision of deposition model results. It provides a foundation of information that can help natural resource trustees decide how to deploy available resources following a spill both across different types of affected areas and within them. It also highlights several components that *do not* need to be assessed immediately following a spill, thereby potentially freeing resources for more time critical data collection efforts.

While the suggestions outlined above will help trustees to proactively manage the level of precision in deposition model results, these components have not been analyzed exhaustively, and there are other sources of uncertainty that have not yet been examined. For example, future research looking at actual deposition patterns following a spill event would help to provide more realistic bounds for the potential for model over- or underestimation when deposition rates are not constant within a search interval. While the bounding simulations conducted as part of this paper suggest that deposition models on average will tend towards overestimation of avian deposition as the search interval increases, these results reflect maximum potential rather than likely overestimation or underestimation arising from use of the constant deposition assumption. This research would also allow for the correction for this type of bias in model results. Such an examination would allow for further refinement of the guidance provided in this paper.
